# Addressing the Gender Divide: Quality of Life and Social Support for Older Men and Women in Rural India

**DOI:** 10.1155/jare/6659622

**Published:** 2026-01-11

**Authors:** Sila Jana, Soumi Paul, Susmita Mondal, Dipak K. Midya, Kapil Dahal

**Affiliations:** ^1^ Department of Anthropology, Vidyasagar University, Midnapore, 721102, West Bengal, India, vidyasagar.ac.in; ^2^ Central Department of Anthropology, Tribhuvan University, Kathmandu, Nepal, tribhuvan-university.edu.np

**Keywords:** gender, older people, quality of life, social support, socioeconomic factors

## Abstract

**Objectives:**

This study investigated gender disparity in quality of life (QOL) and social support among older adults in West Bengal, India.

**Methods:**

The study was conducted among 200 older people. The WHOQOL‐BREF questionnaire and the MSPSS scale were used to measure the respondents’ QOL and social support, respectively. Bivariate correlation and binary logistic regression were performed.

**Result:**

This study showed that being female (AOR: 2.53; CI: 1.32–4.86), low social support (AOR: 5.18; CI: 1.98–13.57), moderate social support (AOR: 4.17; CI: 2.05–8.49), functionally impaired (AOR: 2.25; CI: 1.04–4.86), and being widowed (AOR: 2.95; CI: 1.29–6.73) were significantly associated with poorer QOL of older adults. The interaction effect showed that the QOL of older men would experience a greater improvement than that of older women as a result of increased social support.

**Conclusion:**

Due to inadequate research on the relationship between QOL and social support with special emphasis on gender in India, this study will provide insight for planning interventions to improve older individuals’ QOL.

## 1. Introduction

The global population is aging at an unprecedented rate. By 2030, the worldwide frequency of the older population (60 years and above) will surpass that of the younger population aged below 15 years [1]. Not surprisingly, India has transitioned into an aging society at the end of the twentieth century, and by 2030, the older population is projected to rise from 90 million to 315 million in the country [2]. Typically, the aging population is associated with a rise in infirmity and disability [3, 4]. As people age, they become increasingly reliant on others and relinquish specific roles and responsibilities. This dependency on others may result in a decrease in life satisfaction and quality of life (QOL). An important social risk factor in old age is the narrowing of one’s social circle due to decreased social participation. Being older signifies a vulnerable and risky time in life. Therefore, it is imperative to prioritize this vulnerable section and improve their QOL.

The concept of QOL is now gaining popularity in the field of medical science [5]. The QOL index is a measurable parameter used to assess the requirements and health status of older people [6]. It is considered a key indicator of the efficacy of treatment and care interventions in older adults [7]. The WHOQOL group (1994) defines QOL as “an individual’s perception of their position in life in the context of the culture and value systems in which they live, as well as in relation to their goals, expectations, standards, and concerns.” QOL of older adults has deteriorated in tandem with social isolation, economic dependency, functional deterioration, and autonomy of the younger generations. The paradox of longevity on the one hand, and significantly worsening QOL on the other, is very complex [8]. Inadequate education, poor economic conditions, and lack of social interaction lead to a reduction in the QOL of older people [9].

Prior studies have examined the correlation between various associated factors and the QOL of older individuals. Bhandari et al. [10], based on their study in Kathmandu, Nepal, stated that living alone, poor educational attainment, and the increase in age are positively correlated with lower QOL. A study in China deliberated that older adults experienced a decline in their QOL when they had multiple chronic conditions. This is because of having a higher prevalence of multimorbidity that could worsen the severity of the diseases, resulting in reduced ability to perform daily activities, increased social isolation, and diminished overall well‐being [11]. Additionally, decreased activities of daily living (ADLs), depression, and cognitive deterioration are associated with poor QOL [12–15]. In Bhutan, Dorji et al. [16] observed that the QOL of older people was negatively impacted by the breakdown of intimate familial ties. Kumar et al. [17] revealed better QOL of older people living in joint families in India. Previous research established that QOL in diverse groups was associated with age, gender, socioeconomic condition, education, physical activity, chronic disease, and social support [7, 18–20]. Among these predictors, social support was a key factor that was found to be associated with QOL [20, 21].

Social support is an interactive process where the network members provide financial, emotional, and instrumental support to each other, and this dominant factor plays a key role in predicting the health and well‐being of children and of older people [22]. As social support plays a protective role in connection with the physical and psychological well‐being of elderly people, an absence of social support has certain negative impacts on older people. Familial support offers psychological benefits that aid individuals in reducing their levels of stress and despair, increasing self‐esteem, and enhancing cognitive function. Besides family support, peer support plays a crucial role in the well‐being of older adults. Receiving social support from others fosters a sense of being cared for, respected, and connected within a network of communication and mutual responsibility [23, 24]. In contemporary societies, where the population is continuously aging, social support appears to play a decisive role in maintaining a good standard of health and QOL for older adults [25]. A similar observation was made in a study conducted in China [26].

QOL is significantly shaped by gender roles, particularly through the dynamics of giving and receiving social support. Gender‐based differential treatment influences not only access to support but also the building of decision‐making power and perception of health across different countries and cultures [27–29]. For instance, Hsu [30] found that older Taiwanese women experienced a poor QOL. A study in Austria demonstrated that gender influenced the health‐related QOL of individuals depending on age [31]. Mathud et al. [32] have established an indisputable correlation between gender, social support, and well‐being of elderly individuals. Additionally, older men and women do not play similar social responsibilities in society; hence, gender inequalities in well‐being may be anticipated [33]. Lee et al. [28] demonstrated that older male individuals reported a higher QOL than their female counterparts. Hence, it is essential to investigate the effects of social support on QOL of older individuals, with a particular emphasis on gender differences, as studying the impact of social support on QOL among older adults, particularly in terms of gender disparities, has been a neglected area [34, 35], especially in India. There is no study available that established the association between the three variables, namely QOL, social support, and gender.

Numerous investigations examined the factors that may influence social support and QOL in many nations [36, 37]. There is limited focus on how gender influences the QOL and access to social support in developing countries. Nevertheless, evidence is scarce regarding such investigations in India. To be more specific, there is a dearth of research that examines gender disparities in the relationship between QOL and social support among elderly people in India. The patriarchal family structures in India tend to marginalize older women, especially widows; thus, they experience financial and social insecurity. Moreover, India is experiencing swiftly shifting social forces, such as the disintegration of joint families and mounting economic demands on families, which affect the conventions of caregiving. Under this backdrop, in this study, we endeavored to examine the relationship between QOL and social support, hypothesizing that a lower level of social support is associated with a lower level of QOL. Additionally, this study aimed at investigating gender disparities in connection with social support and QOL among older individuals in India. Such study will be useful for policymakers to implement gender‐sensitive public health policies, particularly in connection with elder care services, retirement planning, and social programs to reduce gender‐specific vulnerabilities.

## 2. Research Methodology

### 2.1. Area and People

This paper is based on our community‐based cross‐sectional study that was conducted across three villages in the Paschim Medinipur District of West Bengal, India. The villages, namely *Amratala, Phulpahari,* and *Golapichak*, with similar socioeconomic status, were randomly selected out of the villages in the Midnapore Sadar Block. The sample size of the study was calculated by using the following formula:

(1) n=z2pqd2=1.962∗0.150.85∗0.052=19610+% non−response rate=216,

where *n* = desired sample size, *z* = level of confidence interval at 95%, *p* = prevalence of low QOL from previous studies in India (15.3%), *q* = 1 − *p*, and *d* ^2^ = degree of accuracy desired, usually set at 0.05.

The sample size was estimated to be 216. Among 216 respondents, 3 older individuals were too mentally ill to respond properly, 8 individuals could not be contacted as they were admitted to the hospital and were out of station, and 5 people withdrew themselves from the interview process. Hence, the final sample size became 200. Respondents were recruited through a household survey (i.e., door‐to‐door visit) in the selected study areas. Data collection was facilitated by an extensive questionnaire that was pretested among 15% of the study participants. Respondents who were either unwilling to participate in the study or had severe mental and physical health issues were excluded. The questionnaire was initially prepared in the English language and then translated into the local language, that is, Bengali, with the linguistic experts, and then back translation was carried out to maintain the semantic equivalence.

We explained the purpose of this research to the respondents and assured them that the information to be gathered would be kept private, would be used exclusively for research, and would only be utilized anonymously. The respondents were informed that they could discontinue their participation in the study at any time during or after the interview. Before the interview, an informed consent form was either signed or fingerprinted by every respondent following institutional human ethics protocol. Using numerical coding, all participants’ privacy and anonymity were protected at every stage of the research.

### 2.2. Measurement of the Variables

Social support of elderly people was measured for this study through the Multidimensional Scale of Perceived Social Support (MSPSS). The MSPSS is a validated multidimensional scale to determine the adequacy of social support that the respondents received from their families, friends, and significant others [38]. This scale focuses on how each individual perceives the socioemotional assistance they receive from the aforementioned sources. There are 12 questions in the MSPSS tool, with 4 items in each of the 3 subscales. To evaluate each question, a seven‐point Likert scale was used, with a range of 1 (“extremely strongly disagree”) to 7 (“extremely strongly agree”). All the scores were added up and divided by 12 to obtain the final score that ranged between 1 and 7. The perceived social support was confirmed by a higher score. The mean score ranging from 1 to 2.9 represented “poor social support”; scores ranging between 3 and 5 were considered “moderate social support,” and scores between 5.1 and 7 represented “high social support” [39]. The reliability of the MSPSS scale was good with Cronbach’s alpha = 0.93 in a previous study [40]. The Cronbach alpha of this scale was 0.787 in this study.

The QOL was examined by using the WHOQOL‐BREF questionnaire, a shorter version of WHOQOL‐100 [41]. It was a self‐administered questionnaire consisting of 26 questions across four domains on the perception of older people about their health and well‐being over the previous 2 weeks. Questions were answered on a five‐point Likert scale, with 1 denoting disagree and 5 denoting completely agree. The higher score (130) is denoted as “high quality of life,” and the lower score (26) is denoted as “poor quality of life.” The scores ≤ 60 denoted low‐level QOL and the scores > 60 denoted high‐level QOL of the respondents [42]. The QOL scale showed good reliability with Cronbach’s alpha = 0.91 in a previous study [43]. The Cronbach alpha of this scale was 0.97 in this study.

Data regarding socioeconomic variables, namely age, gender, community, marital status, occupation, and living arrangement, were collected. Family income (monthly) was categorized as low (25th percentile: INR 9000), middle (26th to 74th percentiles: INR 9001‐21999), and high (INR ≥ 22,000) income quarterly. Functional capacity was measured using the ADL scale [44].

### 2.3. Analysis of Data

The characteristics of the respondents and the distribution of their QOL were illustrated through descriptive statistics. To examine the relationship between variables, bivariate correlation was adopted. The linearity of the correlation between the potential factors was evaluated using the Spearman and Pearson coefficients. Furthermore, binary logistic regression models were employed to explore the factors that affected the QOL of older adults. Model 1 was adjusted for gender and social support, and Model 2 for gender, social support, age, education, occupation, living status, marital status, functional impairment, gender of the main provider of support before, and family income level, whereas Model 3 focused on examining the interaction effect. To examine the gender‐specific disparities, the interaction of social support with gender was included in Model 3. Data analyses were carried out using SPSS (version 25.0).

## 3. Results

The socioeconomic profile of people under study is documented in Table 1. The average age of the study respondents was 70.16 (SD = 8.8), with 49.5% of the older respondents aged 60–69 years, 28.5% aged 70–79 years, and 22% aged 80 years and above. Forty‐nine percent of people were male, and the rest were female. A total of 53.5% of respondents were currently married, and 47.5% of respondents were categorized as widows/widowers. With regard to education, 64.5% had no formal education, 31.5% had upper primary education, and only 4% had a secondary education and above. In terms of monthly family income, 27.5% of respondents had low family income, 45.5% had medium family income, and only 15% had high family income. Table 1 also demonstrates the socioeconomic profile of both older male and female respondents. This table further demonstrates the gender differences in some socioeconomic characteristics (viz. ethnic orientation, marital status, occupation, living arrangement, and family income). Interestingly, older male respondents had higher QOL scores than their female counterparts. Additionally, older men had better social support than older women.

**Table 1:** Socioeconomic profile of study people.

Variable	Total		Male		Female		*χ* ^2^/*t*‐test
	*N* (%)	Mean (SD)	*N* (%)	Mean (SD)	*N* (%)	Mean (SD)	
QOL		71.2 (16.73)		75.2 (15.18)		67.4 (17.30)	2.384^∗^
Social support		36.1 (14.3)		39.1 (14.03)		33.1 (14.11)	2.246^∗^
Age		70.1 (8.84)		69.2 (7.45)		71.03 (9.95)	−0.519
Ethnic group							5.122^∗^
Tribe	100 (50)		41 (41.8)		59 (57.8)		
Nontribe	100 (50)		57 (58.2)		43 (42.2)		
Marital status							34.030^∗∗∗^
Currently married	107 (53.5)		73 (74.5)		34 (31.8)		
Widow/widower	93 (46.5)		25 (25.5)		68 (66.7)		
Educational status							4.444
No formal education	129 (64.5)		57 (58.2)		72 (70.6)		
Up to upper primary	63 (31.5)		35 (35.7)		28 (27.5)		
Secondary and above	08 (4.0)		06 (6.1)		02 (2.0)		
Occupation							7.154^∗∗^
Not working	58 (29)		61 (62.2)		81 (79.4)		
Working	142 (71)		37 (37.8)		21 (20.6)		
Living arrangement							5.099^∗^
Alone	170 (85)		09 (9.2)		21 (20.6)		
Coresiding	30 (15)		89 (90.8)		81 (79.4)		
Family income							2.454
Low	55 (27.5)		27 (27.6)		28 (27.5)		
Medium	91 (45.5)		40 (40.8)		51 (50)		
High	54 (27)		31 (31.6)		23 (22.5)		

^∗^ *p* < 0.05.

^∗∗^ *p* < 0.01.

^∗∗∗^ *p* < 0.001.

**Figure 1:**
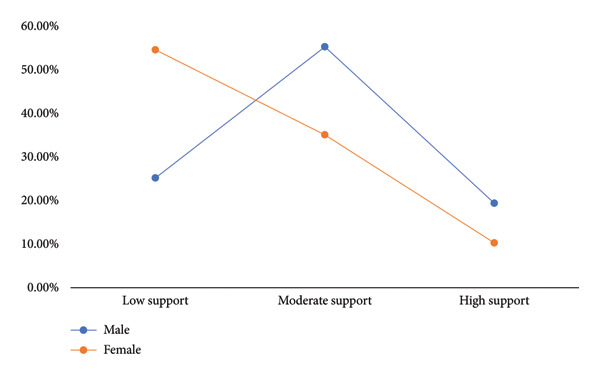
Social support among older male and female respondents.

Figure 1 depicts the prevalence of social support among male and female respondents. It showed that the majority of the female respondents had low social support, with 35.10% having moderate support and 10.30% having high support. Among the male respondents, 25.2% had low social support, with 55.30% having moderate social support and 19.40% having high social support.

Table 2 displays the results of the correlation analysis. Social support was positively correlated and gender was negatively correlated with QOL among the older people. Among the control variables, age was negatively correlated with QOL among older respondents. With aging, the respondents had worsened conditions of QOL. Other factors such as occupation, living arrangement, and family income were positively correlated with QOL. Marital status was found to be negatively correlated with QOL among the respondents.

**Table 2:** Bivariate correlation among potential factors for QOL among older people.

	1	2	3	4	5	6	7	8	9	10
QOL (1)	1									
Age (2)	−0.311^∗∗∗^	1								
Social support (3)	0.487^∗∗∗^	−0.098	1							
Ethnic group (4)	−0.076	0.075	0.135	1						
Gender (5)	−0.173^∗^	−0.114	−0.167^∗^	−0.210^∗∗^	1					
Marital status (6)	−0.258^∗∗∗^	0.004	−0.154^∗^	−0.090	0.379^∗∗∗^	1				
Education (7)	0.123	0.066	0.142^∗^	0.330^∗∗∗^	−0.142^∗^	−0.140^∗^	1			
Occupation (8)	0.348^∗∗∗^	−0.231^∗∗∗^	0.132	−0.132	−0.157^∗^	−0.132	−0.007	1		
Living arrangement (9)	0.306^∗∗∗^	0.017	0.182^∗∗^	−0.252^∗∗^	−0.097	−0.366^∗∗∗^	0.003	0.083	1	
Family income (10)	0.270^∗∗∗^	0.004	0.047	0.065	0.119	0.044	0.008	0.026	0.031	1

^∗^ *p* < 0.05.

^∗∗^ *p* < 0.01.

^∗∗∗^ *p* < 0.001.

### 3.1. Factors Associated With QOL

Table 3 shows the regression analysis of socioeconomic risk factors for QOL among older people. Three models were performed in this table. Model 1 controlled for the gender and social support of the older respondents. In Model 1, female respondents had 2.53 times poorer QOL, compared to their male respondents (AOR: 2.53; CI: 1.32, 4.86). Additionally, respondents with low social support had 5.18 times (AOR: 5.18; CI: 1.98, 13.57) and moderate social support was 4.17 times (AOR: 4.17; CI: 2.05, 8.49) greater odds to have lower QOL, compared to the respondents who had high social support.

**Table 3:** Regression analysis of factors associated with QOL of the older population.

Characteristics	Model 1	Model 2	Model 3
	AOR (95% CI)	AOR (95% CI)	AOR (95% CI)
Gender			
Male	Ref	Ref	
Female	2.534^∗∗^ (1.320–4.862)	3.384^∗∗^ (1.421–8.059)	
Social support			
Low support	5.186^∗∗∗^ (1.981–13.577)	5.055^∗∗∗^ (2.088–12.239)	
Moderate support	4.174^∗∗∗^ (2.052–8.493)	4.401^∗∗∗^ (1.314–14.738)	
High support	Ref	Ref	
Age			
Young–old		Ref	Ref
Middle–old		2.469 (0.842–7.243)	3.254^∗^ (1.117–9.483)
Oldest–old		6.149^∗∗∗^ (2.016–18.757)	7.117^∗∗∗^ (2.380–21.282)
Education			
No formal education		5.039 (0.823–30.851)	6.661^∗^ (1.123–39.527)
Up to upper primary		2.744 (0.462–16.293)	3.357 (0.584–19.282)
Secondary and above		Ref	Ref
Occupational status			
Working		Ref	Ref
Not working		1.554 (0.676–3.570)	1.340 (0.593–3.026)
Marital status			
Married		Ref	Ref
Widow		2.950^∗∗^ (1.292–6.736)	2.800^∗∗^ (1.323–5.928)
Living arrangements			
Coresiding		Ref	Ref
Alone		2.124 (0.613–7.363)	1.739 (0.539–5.616)
Functional status			
Impaired		2.250^∗^ (1.045–4.846)	2.441^∗^ (1.172–5.085)
No impaired		Ref	Ref
Community			
Tribe		Ref	Ref
Nontribe		1.624 (0.642–4.106)	1.225 (0.509–2.950)
Monthly family income (INR)			
Low		2.963^∗^ (1.119–7.845)	2.473 (0.942–6.494)
Medium		1.737 (0.739–4.086)	1.789 (0.755–4.138)
High		Ref	Ref
Gender of the main provider of support			
Male		Ref	Ref
Female		2.127 (0.632–7.159)	1.558 (0.501–4.848)
Gender # social support			
Male # high support			Ref
Male # moderate support			0.758 (0.209–2.742)
Male # low support			5.736^∗∗^ (2.256–14.580)
Female # low support			9.579^∗∗∗^ (3.582–25.620)
Female # moderate support			6.316^∗∗^ (1.791–22.278)
Female # high support			0.951 (0.181–4.993)
Pseudo − *R* ^2^	0.379	0.454	0.438

^∗^ *p* < 0.05.

^∗∗^ *p* < 0.01.

^∗∗∗^ *p* < 0.001.

Model 2 also demonstrated that the older women had higher odds of having poor QOL (AOR: 3.38; CI: 1.42, 8.05). Similarly, respondents with low (AOR: 5.05; CI: 2.08, 12.23) and moderate (AOR: 4.40; CI: 1.31, 14.73) social support had poor QOL, compared to the respondents with high social support. Furthermore, respondents aged ≥ 80 years (AOR: 6.14; CI: 2.01, 18.75) significantly had lower QOL. Interestingly, widow respondents had 2.95 times higher odds of having lower QOL than the respondents who were currently married (AOR: 2.95; CI: 1.29, 6.73). Again, the older people from low family income had poorer QOL than the older people from high family income (AOR: 2.96; CI: 1.19, 7.84). Furthermore, functionally impaired older adults had higher odds of having poor QOL than those who were not functionally impaired (AOR: 2.25; CI: 1.04, 4.84).

Model 3 was performed to examine the interaction effect of gender and social support of the older respondents on their QOL. Older men who had low social support had poorer QOL than older men who had high social support (AOR: 5.73; CI: 2.25, 14.58). Female respondents who had low (AOR: 9.57; CI: 3.58, 25.62) and moderate (AOR: 6.31; CI: 1.79, 22.27) social support showed higher odds of having lower QOL than the male respondents who had high social support.

## 4. Discussion

Aiming at establishing the relationship between gender disparity, QOL, and social support among older adults, this study demonstrated that age was negatively correlated with QOL. As people age, the level of dependency is found to increase among older individuals, which could lead to a decline in their QOL [45]. The individuals aged 60–69 years exhibited higher QOL compared to the other two age groups. The older adults aged 60–69 years were still in their early phases of physical deterioration, although physical health could be impaired with age. Older individuals of advanced age exhibited a greater level of independence in their daily activities. This could potentially enhance their QOL. This study also demonstrated a significant correlation between social support and QOL among the older participants. It was found that the greater the social support among older respondents, the greater their QOL. Similar results were documented in the studies conducted in India [46] and in other countries [45, 47–49]. It was also found in this study that women had a higher prevalence of low social support, whereas men tended to have higher social support. Similar findings were reported in the studies undertaken in various developed and developing nations [50, 51]. Previous studies conducted in Middle Eastern nations also yielded comparable findings [21, 52].

Older individuals with inadequate family income exhibited higher odds of experiencing a poor QOL. Our study also indicated that as family wealth rises, the QOL and the level of happiness among older individuals increase. Prior research also reported that there was a positive correlation between household income and the QOL among older adults [53–56]. Elderly people with a lower socioeconomic status are more likely to experience heightened levels of stress and frustration as a result of social comparison and dissatisfaction. However, Kim et al. [57] observed that despite an increase in income, the QOL of older people did not improve. We recommend further research to clarify the relationship between family income and QOL. Functionally impaired older adults had higher odds of experiencing poor QOL, which is aligned with previous studies in China [58] and Poland [59].

Our study determined a strong relationship between the marital status of older adults and their QOL. The older individuals who were married reported a higher QOL than the older individuals who had lost their spouse. Previous research undertaken in India [17, 46, 60] and other countries [45, 49, 61] established a correlation between marital status and the QOL among older individuals. It was indicated that those who were married had superior mental and physical well‐being and a longer lifespan compared to those who were not married [62, 63]. Additionally, married older adults could share and alleviate their spouse’s stress. Furthermore, being married ensured that an individual could receive support and help to prevent loneliness. In addition, fostering a positive relationship with one’s spouse could improve one’s mental well‐being [64]. These variables might improve the QOL of the older married adults compared to their unmarried counterparts.

This study further showed that the older male adults reportedly had higher QOL compared to their female counterparts. Findings from previous studies in India [17, 60], Sri Lanka [49], Vietnam [65], Bangladesh [66], Myanmar [67], Qatar [6], and Malaysia [68] revealed that females tended to have low QOL in relation to their male counterparts. The aforementioned countries in Southeast Asia and the Middle East used to follow certain traditional gender norms that might contribute to the disparity in QOL between males and females in the respective societies. However, no gender disparity in QOL was reported in Japan [69] and Thailand [70]. This consistency may be linked to the variations in cultural values regarding gender across different countries. Significantly, the interaction effect showed gender disparity in QOL among older people. Females with low and moderate social support had higher odds of having poor QOL in reference to men with high social support. Additionally, it was found that men who accessed low social support had poor QOL.

The life of women in rural India is often influenced by poor economic autonomy, unequal distribution of family resources, and increased burden of caregiving roles. They are usually considered automatic caregivers, not as care receivers at the family level and beyond. They are supposed to provide social support to other family members in every odd situation but not vice versa in many cases. Thus, the social support mechanism does not provide women with the same level of protection as it provides to men. Moreover, various patriarchal norms restrict their decision‐making capacity and freedom of mobility. Thus, the women face the very high odds of a poor QOL with low social support.

### 4.1. Limitations

The study had several limitations. The relatively smaller size of the analytic sample (*n* = 200) caused by the exclusion of missing data could have created selection bias because the excluded participants were older and in worse health condition, which may have resulted in a minor overestimation of QOL scores and limited generalizability of results. The study design employed was cross‐sectional, which precluded the establishment of a causal link between the variables. Furthermore, the data for this study were obtained through a structured questionnaire that relied on self‐reported information from the participants. This method might be susceptible to recall bias. Furthermore, the study was conducted in a rural area of India with a limited sample size. Therefore, it may not be generalized to the entire country, though it reflects the trend. In future studies, we will take a larger sample that can provide a representative dataset for the whole nation. Nevertheless, the result of this study seems to possess a relatively high level of reference value in rural India due to plentiful similarities across rural regions in India.

## 5. Conclusion

The study offered crucial insights that might enhance our understanding of the QOL of older people in rural India. One of the primary findings was that the older women consistently reported a lower QOL compared to that of men. The presence of gender disparity in QOL indicated that India requires the implementation of increasingly effective policies, programs, and services to promote gender equity in connection with the QOL. This study also demonstrated a negative correlation between age and QOL. As individuals age, their level of social support decreases, resulting in a decline in achieving their overall QOL. This study further showed that social support had the most significant impact on QOL. Therefore, this study highlights the need for community‐based programs and policies that enhance effective social support mechanisms and improve the QOL for older individuals in India. Socioeconomic factors significantly impact the QOL among older people, as found in this study. Among the socioeconomic determinants, lower income level and being widowed are significantly associated with poorer QOL of older individuals. The results of the current research demonstrated the necessity of determining specific welfare benefits and informing the policy‐making process to increase the QOL of the geriatric population. Moreover, further research is needed to explore other potential factors that may impact the QOL among older individuals and evaluate potential interventions to determine their actual impact.

## Conflicts of Interest

The authors declare no conflicts of interest.

## Funding

The authors received no specific funding for this work.

## Data Availability

As the study is based on the data collected for a doctoral dissertation and the doctoral dissertation is yet to be awarded, the authors would prefer not to share the data for public access, but the data are available from the corresponding author on reasonable request.
